# Implications of environmental toxicants on ovarian follicles: how it can adversely affect the female fertility?

**DOI:** 10.1007/s11356-021-16489-4

**Published:** 2021-10-09

**Authors:** Keerthi Priya, Manjunath Setty, Uddagiri Venkanna Babu, Karkala Sreedhara Ranganath Pai

**Affiliations:** 1 Department of Pharmacology, Manipal College of Pharmaceutical Sciences, Manipal Academy of Higher Education, Manipal, Karnataka 576104 India; 2 Department of Pharmacognosy, Manipal College of Pharmaceutical Sciences, Manipal Academy of Higher Education, Manipal, Karnataka 576104 India; 3 Phytochemistry Department, R & D Centre, The Himalaya Drug Company, Makali, Tumkur Road, Bangalore, Karnataka 562162 India

**Keywords:** Ovarian follicles, Folliculogenesis, PCOS, Multioocytic follicles, Primary ovarian insufficiency, Environmental toxicants

## Abstract

The pool of primordial follicles formed in the ovaries during early development determines the span and quality of fertility in the reproductive life of a woman. As exposure to occupational and environmental toxicants (ETs) has become inevitable, consequences on female fertility need to be established. This review focuses on the ETs, especially well-studied prototypes of the classes endocrine disrupting chemicals (EDCs), heavy metals, agrochemicals, cigarette smoke, certain chemicals used in plastic, cosmetic and sanitary product industries etc that adversely affect the female fertility. Many in vitro, in vivo and epidemiological studies have indicated that these ETs have the potential to affect folliculogenesis and cause reduced fertility in women. Here, we emphasize on four main conditions: polycystic ovary syndrome, primary ovarian insufficiency, multioocytic follicles and meiotic defects including aneuploidies which can be precipitated by ETs. These are considered main causes for reduced female fertility by directly altering the follicular recruitment, development and oocytic meiosis. Although substantial experimental evidence is drawn with respect to the detrimental effects, it is clear that establishing the role of one ET as a risk factor in a single condition is difficult as multiple conditions have common risk factors. Therefore, it is important to consider this as a matter of public and wildlife health.

## Introduction

The story of gametogenesis and sex-specific gonadal formation starts with the origin of pluripotent primordial germ cells (PGC) in the extraembryonic tissue followed by their migration towards the gonadal ridge. This involves various signals between the PGCs, somatic cells of hind gut as well as primordial gonads. Major signaling molecules involved in the maintenance of pluripotency and migration include bone morphogenetic protein (BMP), Wnt3, Kit ligand (KL), etc. (Wear et al. 2016). The type of germ cells that reach these primordial gonads determines gender-specific development. Germ cells undergo mitotic cell division and differentiation to form oogonia that further proliferate synchronously but without proper cytokinesis, to form clusters of cells called germ cell nests or syncytia (Motta et al. 1997; Pepling and Spradling 1998). Within these nests, mitosis is halted, and oogonia differentiate into oocytes. This marks the beginning of meiosis. However, meiosis does not generally proceed beyond the diplotene phase of prophase I. Arrest in prophase I is a consequence of complex signaling processes which only resumes after puberty under the influence of gonadotropins and many other signaling molecules. However, most of the oocytes undergo atresia by programmed cell death, and only a few oocytes sustain (Pepling and Spradling 2001). Nests in which the oocytes are arrested in prophase are eventually invaded by the surrounding somatic cells forming primordial follicles (see Fig. 3). Thus, at the time of birth in humans, two types of structures contribute to the formation of follicle. One, the germ cell that is in the form of primary oocyte and arrested in prophase I which does not develop further until puberty under the influence of hormones. Two, the follicular somatic cells that envelop oocyte which can develop throughout the later follicular stages independent of the oocyte development. Follicular development principally takes place in two phases: the initial gonadotropin-independent phase and the later gonadotropin-dependent phase. The number of primordial follicles at birth is high, and this pool of follicles serves as the ovarian reserve determining the ovarian lifespan and fertility period in a woman’s life. Out of approximately 700,000 primordial follicles formed at birth, 300,000 would be available at puberty and only 400 to 500 would contribute to fertility throughout the reproductive period (Forabosco and Sforza 2007). Since such a drastic reduction in the number of follicles is involved, conservation of follicles by adopting a healthy lifestyle is important. However, exposure to a number of environmental toxicants (ETs) has become unavoidable and inevitable in the present age.

The effect of these ETs on reproductive potential has been a matter of concern off late. A wealth of literature is available that clearly shows detrimental reproductive consequences of various ETs on women’s reproductive health. Different type of ETs like endocrine disrupting chemicals (EDCs), heavy metals, pesticides, cigarette smoke, certain chemicals used in plastic, cosmetic and sanitary product industries can adversely affect female fertility (Barker 2003). Endocrine disrupting chemicals are agents that are found in a variety of consumer products such as cosmetics, agrochemicals, plasticizers and certain foods. Although EDCs were thought to act mainly by nuclear receptors of hormones including estrogen, progesterone, androgen and thyroid receptors, we now understand from the research that they also act on nonnuclear receptors of hormones, receptors of neurotransmitters, orphan receptors and can also be directly alter the steroidogenesis and the hormonal metabolism apart from other reproductive pathways (Diamanti-Kandarakis et al. 2009). EDCs include various chemicals like bisphenol A (BPA), perfluoroalkyl and polyfluoroalkyl substances (PFAs), phthalates, phytoestrogens, polybrominated diphenyl ethers (PBDE), dichlorodiphenyltrichloroethane (DDT), dioxins, perchlorate, triclosan and organophosphates (OP). PFAs and PFOS used for coating surfaces not limited to furniture, leather, clothing, etc. have also shown to affect the female fertility and fecundity over long-term exposure. Many studies have proved the effect of such chemicals on female reproductive system (Gonsioroski et al. 2020; Ding et al. 2020; Vélez et al. 2015). Heavy metals like cadmium, arsenic, mercury and lead have adverse effects on female fertility, and this has been shown in a number of studies (Kumar 2018; Lee et al. 2020; Massányi et al. 2020). Other risk factors like cigarette smoking, accumulation of reactive oxygen species also pose an equal risk in altering the female fertility.

All the above can act as potential risk factors, for several conditions that affect the female fertility and fecundity, especially on a prolonged exposure. Since it is not possible to cover all the effects of every ET on female fertility, this review mainly focuses on the prototypes of the above classes of ETs that have been extensively studied and screened either in vitro, in vivo or in humans for causing fertility-related problems by precipitating polycystic ovary syndrome (PCOS), primary ovarian insufficiency (POI), multioocytic follicles (MOF) and meiotic defects including aneuploidies.

## Environmental toxicants and ovarian follicles

Environmental toxicants (ETs) have been proven to exert detrimental effects on ovaries causing various reproductive problems. One class of such compounds are the EDCs or endocrine disruptors. Most EDCs are man-made chemicals. They are found in routinely and frequently used materials such as agrochemicals, personal care products, plastics, metals and additives or contaminants in certain food (WHO-EDC). EDCs can also be found in drinking water, food, air and diet (Azziz et al. 2009; Wee and Aris 2019). They can interfere with the endocrine system of the body and affect various hormone-related functions. According to the Environmental Protection Agency (EPA), an EDC is defined as ‘an exogenous agent that interferes with biosynthesis, secretion, transport, metabolism, binding action, or elimination of natural blood-borne hormones present in the body, which are responsible for homeostasis, reproduction, and developmental process’ (Diamanti-Kandarakis et al. 2009). EDCs can, not only affect the reproductive system in adulthood, but also form a basis for adult reproductive problems upon exposure during the fetal or developmental phase. Their disruptive action on the endocrine system is often termed as ‘the fetal basis of adult disease’ (Barker 2003) indicating that the manifestation of disease can occur much later in life after the early exposure in the developmental life. Some common EDCs are bisphenol A, PFAs, phthalates, phytoestrogens, PBDE, DDT, dioxins, perchlorate, triclosan, organophosphates, etc (NIEHS-Endocrine Disruptors). Toxicities with EDCs can occur even at environmental concentrations or at lower amounts on chronic exposure. Several clinical studies have identified the presence of EDCs not only in the blood but also in the follicular fluid. In a study by De Felip et al. (De Felip et al. 2004) follicular fluid aspirated from women undergoing multiple ovulation and oocyte retrieval for in vitro fertilization from Rome and Italy in the year 2000 contained pesticides at a level that could affect the biological competence of oocyte. Similarly, significant serum levels of organophosphates were also found in women undergoing assisted reproductive technologies (Meeker et al. 2009). EDCs act in a number of ways as reviewed by La Merrill et al., i.e. by acting as an agonist or antagonist to the hormonal receptors, by altering the expression of receptors, by altering the signal transduction, by interfering with the hormonal synthesis, transport, distribution or clearance, by causing epigenetic changes or by altering the fate of a cell by apoptosis, proliferation or differentiation (La Merrill et al. 2020). Various EDCs like DDT have proven to alter the steroidogenesis in female reproductive system, and their effect was not only found in the gonadotropin signaling pathway but also in other enzymatic and calcium-dependent pathways (Cohn et al. 2003; Craig et al. 2011). Their effects were seen both on theca and granulosa cells of the ovary. Johansson et al*.*(2017) reviewed that there are four important time frames when the ovary is most vulnerable to insults by exogenous agents including EDCs. These stages were stated as gonadal sex determination, meiotic division, follicle assembly and the first wave of follicle recruitment. In a first, evidence to the presence of microplastics was found in the human placenta which were all man-made(Ragusa et al. 2021). Therefore, EDCs can affect the initial and later stages of folliculogenesis along with meiosis.

Similarly, how ETs like cigarette smoke, heavy metals, along with the EDCs can affect female fertility and fecundity by acting as risk factors of various conditions that can alter the processes of follicular development, ovarian steroidogenesis, etc. is discussed in the following sections. However, the reader has to bear in mind that apart from the conditions explained below, ETs are also capable of causing other ovarian insults such as increasing the risk for cancers, birth defects in newborns and spontaneous loss of pregnancy, all of which can be classified as ovarian dysgenesis syndrome (Johansson et al. 2017). As stated earlier, the present paper is focused on the ETs that are well studied for causing four conditions, namely, polycystic ovary syndrome, primary ovarian insufficiency, multioocytic follicles and meiotic defects that are considered main causes for reduced female fertility by directly altering the follicular recruitment, development and oocytic meiosis.

### Polycystic ovary syndrome (PCOS)

PCOS is a complex syndrome, and several definitions are used to explain the condition. However, Rotterdam consensus is a widely accepted criterion, according to which the presence of any two of the following conditions define the manifestation of PCOS: oligoanovulation and hyperandrogenism or polycystic ovaries (PCO)(Broekmans et al. 2006). Biochemical hyperandrogenism is more commonly seen in women with PCOS than clinical hyperandrogenism. Only 80–85% of the women who exhibit clinical hyperandrogenism have PCOS (Azziz et al. 2004, 2009), and certain ethnic groups do not show the symptoms of hyperandrogenism. Hyperinsulinemia is also commonly seen in women with PCOS. Excessive androgen production results from contribution both by ovarian and the adrenal production (Puurunen et al. 2009). The significance of adrenal production of androgens in PCOS was clearly studied. The circulating total and free testosterone and dehydroepiandrosterone sulfate (DHEAS) are found to be elevated in PCOS as a combined effect of both overproduction of the hormones and decrease in the sex hormone binding globulin (SHBG) level (Huang et al. 2010). A huge volume of literature is available to establish the contribution of various EDCs in PCOS. In a case-control study by Vagi et al*.*(2014)(Vagi et al. 2014), it was found that the PCOS group had higher serum levels of two PFAs—perfluorooctanoate(PFOA) and perfluorooctanate sulfonate (PFOS) compared to the non-PCOS controls. In another case-control study by Yang et al*.*(Yang et al. 2015), there was a significant difference in the serum polychlorinated biphenyls (PCB) congeners’ level between PCOS patients and controls indicating the probable role of these organic pollutants in PCOS pathology.

**Fig. 1:**
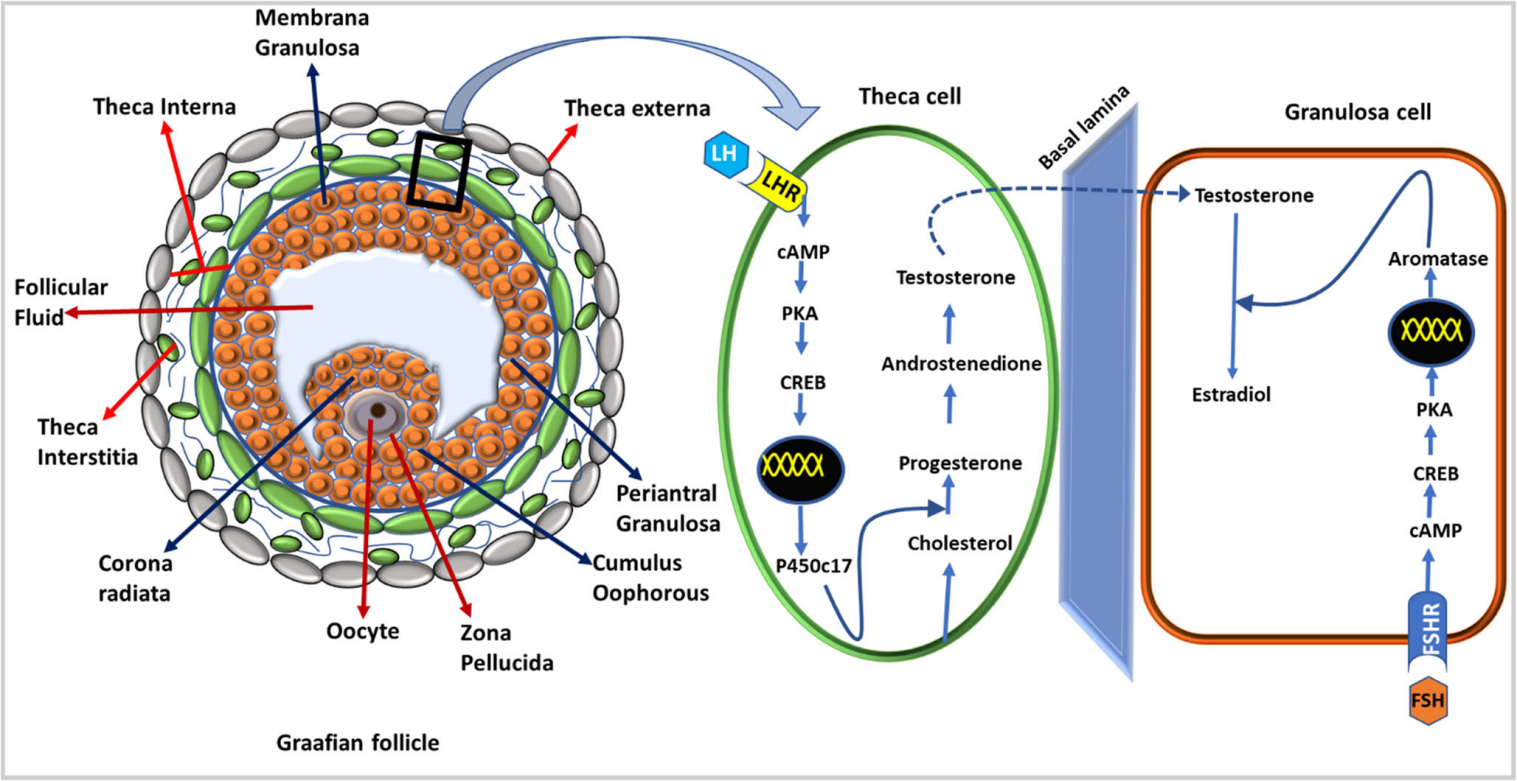
Steroidogenesis in theca and granulosa cells. Dark blue arrows indicate different layers of granulosa cells, and red arrows indicate layers of theca cells. Steroidogenesis occurs in theca cell under the influence of leutinising hormone binding to its receptor and conversion of testosterone by aromatase to estradiol in granulosa cell by the influence of FSH binding to its receptor. In polycystic ovary syndrome, an increased LH action results in increased androgen synthesis causing hyperandrogenism. LH luteinizing hormone, LHR luteinizing hormone receptor, FSH follicle stimulating hormone, FSHR follicle stimulating hormone receptor, cAMP cyclic adenylyl monophosphate, PKA protein kinase-A, CREB response element binding protein

EDCs can contribute to all the important aspects of the syndrome, i.e. increased levels of androgens, anovulation and formation of polycystic ovaries. Many studies have explored the effect of EDCs and increased androgen levels which play a key role in the pathogenesis of PCOS. A generalized hyper functioning of theca cells and hypofunctioning of granulosa cells leading to hyperandrogenism were seen in the pathology of PCOS (Nelson et al. 2001). Anovulation due to hyperandrogenism within ovaries is called functional ovarian hyperandrogenism (FOH). The spectrum of FOH in PCOS and in asymptomatic women with polycystic ovarian morphology is explained by Rosenfield and Ehrmann (Rosenfield and Ehrmann 2016). EDCs causing increased levels of testosterone were reported by Wojtowicz et al. (Wójtowicz et al. 2007) in porcine cells. Steroidogenesis in theca and granulosa cells is depicted in Fig. 1. Numerous studies showed that post-natal exposure of rats and mice to testosterone and androstenedione caused anovulation or ovulatory dysfunction with either normal or delayed puberty (Edwards 1971; McDonald and Doughty 1972; Lookingland et al. 1982; Abbott et al. 2005). With increasing concentrations of mono, ortho PCBs, decreased probability of ovulation was observed (Gallo et al. 2016). The exact mechanism of anovulation unrelated to ET-induced hyperandrogenism has not been perfectly established in the literature. A positive relation was observed between BPA and increased androgen levels in biological fluids of women with PCOS indicating that BPA can raise androgen level. The same trend was observed in adolescents with PCOS (Takeuchi et al. 2004; Kandaraki et al. 2011). BPA has been shown to hyperstimulate the theca cells to produce androgens by dysregulating the key enzyme of steroidogenesis, 17a-hydroxylase(Zhou et al*.*^2008^). Phthalates were also found to stimulate steroidogenesis in ovaries in mouse (Gunnarsson et al. 2008).

**Fig. 2:**
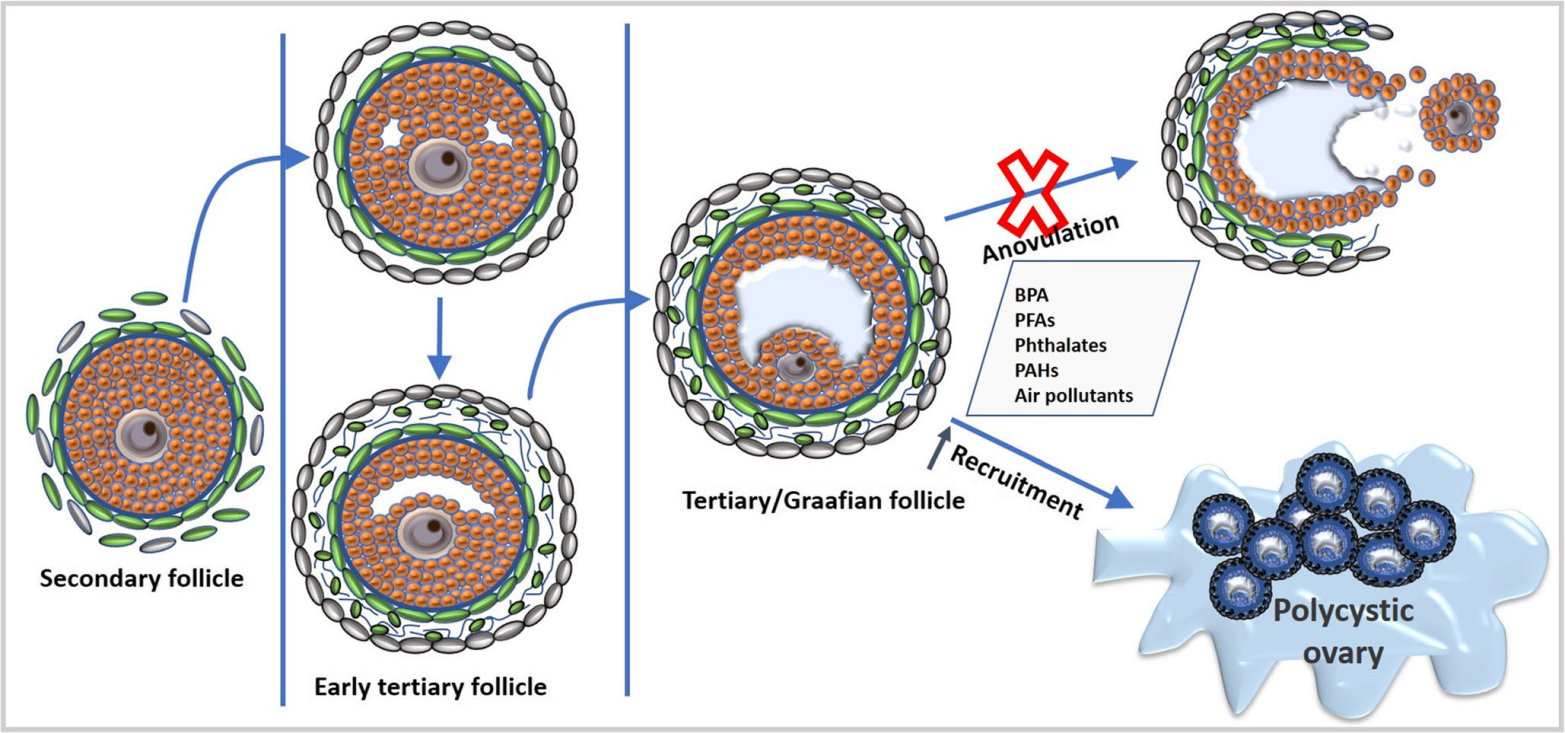
Development of secondary follicle into Graafian follicle with a gradual increase in the antral size. Environmental toxicants alter the steroidogenesis in ovaries causing hyperandrogenism that results in anovulation and accelerated recruitment of follicles culminating in polycystic ovaries

It is also interesting to note that hyperandrogenism also leads to anovulation and polycystic ovaries. During folliculogenesis, recruitment of dominant follicle from the pool of primordial follicles allows the dominant follicle to further proceed to later stages as shown in Fig. 2. However, in women with PCOS, there was recruitment of more than one follicule that resulted in polycystic ovaries. In studies on animals treated with androgens, there was an increased follicular recruitment leading to an increased accumulation of both preantral and antral follicles in ovaries resulting in polycystic ovaries (Hughesdon 1982). This also draws the attention to the fact that EDCs not only affect the hormone-dependent antral follicles, but also hormone-independent stages of follicular development. It is in consensus with the fact the levels of growth differentiation factor 9 (GDF9), KL and anti-mullerian hormone (AMH), which are crucial in the preantral follicle development, were also found higher in PCOS. Androgen treatment also increased the expression of follicle stimulating hormone (FSH) and leutinising hormone (LH) receptors on granulosa cells of anovulatory follicles. Hypersecretion of LH indicates the role of EDCs in dysregulation of both hormone-dependent and independent phases of folliculogenesis (Willis et al. 1998; Weil et al. 1999). This is supported by other studies which also showed increased levels of LH, LH/FSH, fasting insulin, testosterone and DHEAS in PCOS cases who additionally had an elevated level of o,p′-DDT. What is more alarming is that EDCs can also cause epigenetic changes in the DNA of female reproductive system that can affect the future generations in carrying possible PCOS phenotypes (Rutkowska and Diamanti-Kandarakis^2016^). Overall, EDCs can disrupt both hypothalamic-gonadal hormonal regulation along with exerting local paracrine and autocrine mechanisms which can ultimately lead to pathogenesis of PCOS as shown in Fig. 2.

Smoking and exposure to cigarette smoke are also positively correlated with the incidence of PCOS in several studies. In a comparative study between the oligo-anovulatory women with PCOS, women with normal anovulation in PCOS and healthy controls, it was found that smoking was associated with ovulatory dysfunction in a dose-dependent manner (Zhang et al. 2020). However, in this study, both active and passive smoking were considered as criteria for smoking. An oxidative state with decreased antioxidant levels is reported in PCOS. An inflammatory state with increased mononuclear cells and mitochondrial dysfunction with decreased GSH and oxygen consumption is well documented (Palacio et al. 2006; Victor et al. 2011). All of these can be related to smoking. All these inflammatory stimuli can in turn cause steroidogenesis in theca cells by altering enzymes involved in the process. Polycyclic aromatic hydrocarbons (PAHs) are a class of compounds found in cigarette smoke. A study has reported positive correlation between PAHs and the risk of PCOS (Yang et al. 2015). A registry-based national cohort study showed an association between maternal smoking during pregnancy and an increased incidence of PCOS in their daughters during the later life (Valgeirsdottir et al. 2019). Some studies have also reported the role of smoking in aggravating symptoms associated with PCOS. PAHs are also produced from burnt coal, wood, gas, garbage and meat cooked at high temperatures. Therefore, air pollution is also an important source of PAHs.

Interestingly, the role of air pollution was also explored in PCOS. A nationwide population-based cohort study was conducted in Taiwan taking two databases. One was longitudinal health insurance data, and the other was of the air quality data. Concentration of air pollutants such as nitrogen oxides, nitrogen monoxide, sulfur dioxide, nitrogen dioxide and particulate matter (PM) 2.5 were assessed. The study reported that women exposed to the pollutants were at a higher risk of PCOS (Lin et al. 2019). An increase in inflammatory mediators in women exposed to various air pollutants could alter the normal steroidogenesis like in smoking.

### Multioocytic follicles

**Fig. 3:**
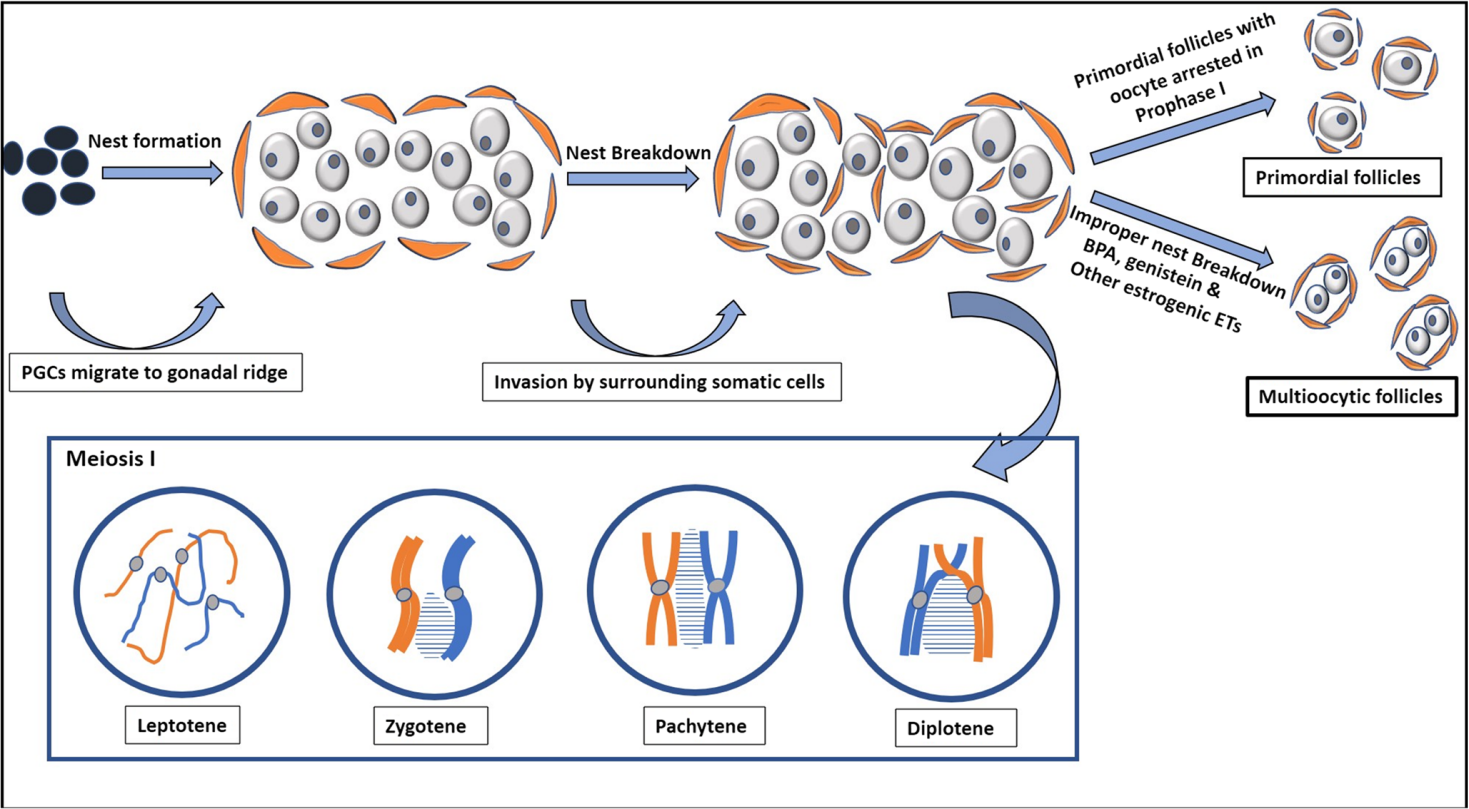
Formation of primordial follicles from PGCs. The PGCs migrate to the gonadal ridge, where they proliferate and differentiate to form nests of oogonia. Within the nests, the oogonia form oocytes which initiate meiosis but are arrested in the diplotene phase of Meiosis I. The surrounding pregranulosa cells invade the nests thus giving rise to primordial follicles. BPA, genistein and other estrogenic environmental toxins (ETs) cause the formation of multioocytic follicles by inhibiting the nest breakdown

Primordial follicle forms when surrounding granulosa cells invade the nests to form single unique follicle with one oocyte surrounded by a layer of flattened granulosa cells (Fig. 3). Hence, only one oocyte resides inside a primordial follicle. But in some cases, two or more oocytes are enveloped by granulosa cells within a single follicle forming multioocytic follicle (MOF). Naturally, MOFs are larger than the normal follicles at the same developmental stage and appear elongated (Telfer and Gosden 1987). The advent of MOF is considered common in many species like bats, rats, mice, dogs, monkeys and humans. Nevertheless, the common presence of MOFs is not to be considered harmless, at least when it occurs in high numbers. In most instances where the frequency of MOFs is high, it can result in the formation of teratomas (Ashley 1973). This hypothesis that ovarian teratomas are a result of fusion of ova has been tested by (Muretto et al. 2001), after observing that the ovarian tissue adjacent to teratomas were occasionally biovulatory, meaning two oocytes were present in a single follicle. The authors have then investigated the removed teratomas of 31 patients with mature teratomas. Among the 31 samples observed, 26 residue ovarian tissues had biovularity. Twenty-four ovaries had ‘hourglass-shaped’ primary follicles with two ova, probably as a result of coalescence. But out of the 30 control samples, only one ovary had biovularity indicating that biovularity is associated with teratomas (Muretto et al. 2001).

Oocyte number in each MOF and the frequency (number of MOF observed/total number of follicles) of MOFs throughout the reproductive life differ from species to species (Silva-Santos et al. 2018). There are many differences beyond just the physical appearance between a unioocyte follicle (UOF) and an MOF. For instance, the quality of fertilized oocyte (if fertilized) from MOF is lower than a fertilized oocyte from a UOF. When examined in vitro, the number of embryos formed from MOF is found to be significantly lower compared to UOF along with a decreased fertilization capacity in porcine ovaries (Stankiewicz et al. 2009). Formation of MOF is attributed to abnormalities in the nest breakdown. As stated earlier, several factors are responsible for nest breakdown as shown in Fig. 3. Therefore, any agent that alters these factors could potentially interfere with MOF formation. The exact causes of MOF formation are not fully established in the literature but compounds possessing estrogenic activity could result in MOF. A lot of literature is available in relation to estrogen and progesterone and the formation MOF. In mice, as germ cell nest breakdown occurs after birth, it is understood that the decrease in the level of estrogen and progesterone at birth is responsible for the formation of primordial follicle. Additionally, studies have also shown that in neonatal mouse, supplementation of estrogen, progesterone and genistein caused inhibition of nest breakdown which was evident in the significant decrease in percentage of single oocytes and assembly of primordial follicles. This was found to be true both in vitro and in vivo indicating progesterone, estrogen and estrogenic substances potentially inhibit the nest breakdown and primordial follicle assembly (Chen et al. 2007). However, since this process does not occur after birth in humans but occurs during the gestation, confusion arose about how primordial follicles form even in the presence of hormones. It is now evident that there is a decreased fetal circulation of hormones during the period when primordial follicles form. Therefore, it is probable that a decrease in the hormonal exposure could result in nest breakdown and primordial follicle assembly. The same has also been demonstrated in fetal tissue of monkey (Thau et al. 1976). This hypothesis is supported by another study where the authors administered aromatase inhibitor to baboons during the nest breakdown and observed that it inhibited the process and more cells remained in the nests (Zachos et al. 2004).

BPA has been shown to induce MOF in various species. In mice, when treated perinatally and investigated, BPA induced polyovular follicles (Suzuki et al. 2002). Another study reported that when BPA was administered, fetal ovary of rhesus monkey had a significant increase in the number of MOF, especially with a single daily dose. Similarly, in case of rodents, the effect was observed both in the increase of MOF and the average number of oocytes in them (Hunt et al. 2012). Similar results were seen in neonatal follicle dynamics of lamb after BPA administration (Rivera et al. 2011). Some studies have indicated that the effect of estrogen on MOF formation is by influencing the activin signaling. It has been shown that estrogen suppresses the expression of activin in neonatal mice thereby affecting the primordial follicle formation resulting in MOF (Kipp et al. 2007). The same is supported by another study in which exogenous activin could increase the number of primordial follicles (Bristol-Gould et al. 2006). Since activin is inhibited by follistatin, it has also been shown to influence the primordial formation by regulating the breakdown of nest cell (Kimura et al. 2011).

### Primary ovarian insufficiency

The reserve of follicles for life is dependent on the endowment of a primordial follicle pool. This follicular reserve determines a woman’s subsequent reproductive potential in the later life. Any attrition in the pool of primordial follicles can adversely affect the reproductive function and could possibly lead to infertility. Insufficient number of follicles at the time of birth or decrease in the number of follicles rapidly at birth or after puberty is called diminished ovarian reserve (DOR). DOR can lead to primary ovarian insufficiency. Various terms like premature ovarian insufficiency, premature ovarian failure and premature menopause are used synonymously with primary ovarian insufficiency (POI). However, more accurate term for the condition is primary ovarian insufficiency (Welt 2008). In primary ovarian insufficiency, there can be an intermittent or occasional ovulation and a regain of follicular function. This means pregnancy is possible unlike in other cases where a complete halt in menstruation/ovulation happens. In woman having POI, there can still be chances of conceiving before attaining a premature menopause below the age 40. The etiology of POI can be attributed to disturbances within the ovaries. It is mainly characterized by hypergonadotropic hypogonadism, amenorrhoea and attrition in the follicular reserve. Diagnosis can be made based on the following characters: sex hormone deficiency, two confirmatory readings of FSH levels more than 40 IU/L with a gap of at least a month, amenorrhoea for not less than 4 months in women of age less than 40 years (De Vos et al. 2010). AMH and inhibin B levels are also found to be altered (Knauff et al. 2009), making AMH an additional screening test although not confirmatory. AFC is an important non-invasive diagnostic parameter for DOR to determine the antral follicle count in ovaries. As DOR can also result in POI, it can serve as a futuristic diagnosis for POI. Since POI exhibits not a single, but a myriad of features, it is likely that other conditions could manifest as well including osteoporosis, cardiovascular and neurological problems. A number of genes are associated with POI, and recent studies have established the genetic defects of the implicated genes in POI (Yatsenko and Rajkovic 2019). POI can be spontaneous or induced. ETs are one of the causative factors of induced POI, and it is not until recently that the role of ETs is being explored in this regard.

**Fig. 4:**
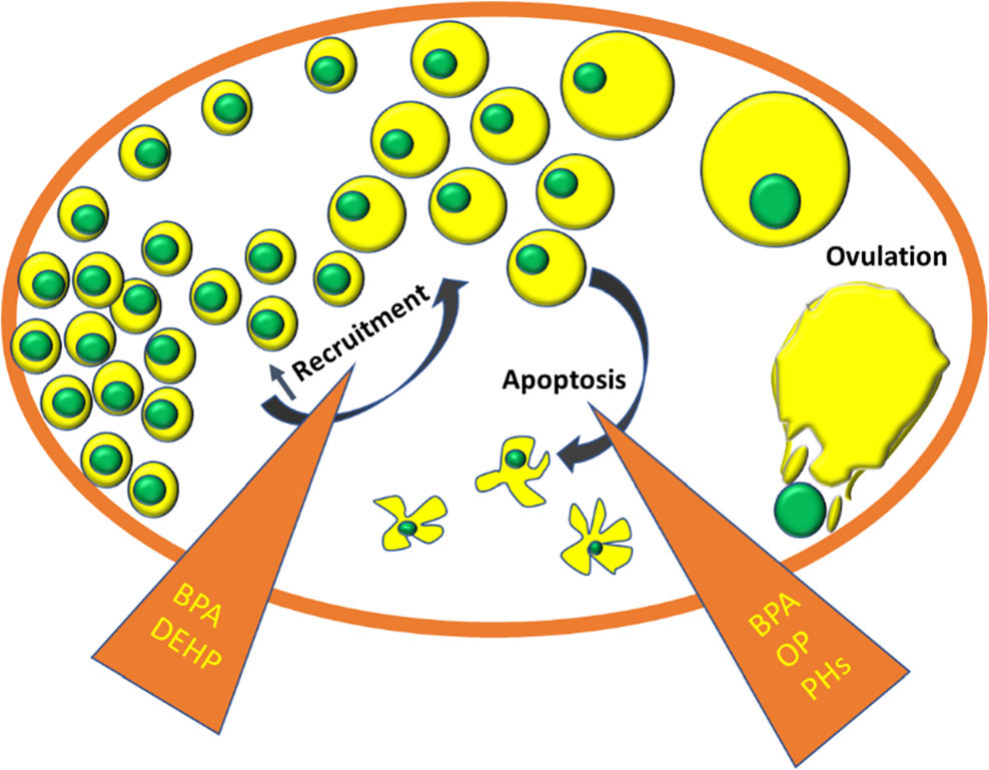
Chemicals causing primary ovarian insufficiency by accelerating the recruitment of ovarian follicles and follicular apoptosis. BPA bisphenol A, DEHP diethylhexyl phthalates, OP organophosphates, PAHs polycyclic aromatic hydrocarbons

Various environmental factors are recently found to interfere with the ovarian function causing POI. ETs have shown to deplete the ovarian reserve and the depletion is linked to prenatal, postnatal and later stages of life. It has also been observed that the most sensitive stage of follicular development with environmental toxicant exposure is variable (Dominguez et al. 2016). ETs can induce diminution of the ovarian follicular reserve (Fig. 4) primarily by acting on estrogen receptors (ER) and arylhydrocarbon receptors (AhR)(Beischlag et al. 2008; Shanle and Xu 2011). This could be through oxidative stress (Luderer 2014)and/or by epigenetic modifications (Vabre et al. 2017). Both these receptors are known to play a pivotal role in early folliculogenesis. However, there is an argument around ER as to whether they are important in the early phase before primordial follicle formation or not. Some research has shown that estrogens do interfere in the primordial follicle formation (Fowler et al. 2011). The role of both ER alfa and beta in ovaries are well explored. ER alfa is highly expressed on theca cells and ER beta on granulosa cells. While the former is essential in ER mediated local effects resulting from FSH and LH stimulation, the latter is important in HPA feedback and steroidogenesis (Couse et al. 2005; Woodruff and Mayo 2005; Armenti et al. 2008). AhRs are expressed on follicles and granulocytes at all the stages of follicular development (Robles et al. 2000). Recent studies have shown that AhR plays a crucial role in the apoptosis of early follicles by inducing pro-apoptotic factor Bax resulting in follicular atresia (Pru et al. 2009; Craig et al. 2011). The role of oxidative stress and the damage rendered by reactive oxygen species (ROS) on the ovarian reserve resulting in POI has been shown by some studies. A cross-sectional case-control study was conducted which showed a significant level of certain ROS in women with POI compared against the healthy controls (Tokmak et al. 2015).

Likewise, triclosan (TCS), a chlorinated aromatic compound used as a broad-spectrum antibiotic in cosmetics, veterinary, plastic industries and household products, was found to affect the ovarian reserve in women. It is considered as one of the toxicants present in water. Although its action on skin from cosmetic products is short, after it is released into the sewage water, it cannot be completely removed through wastewater treatment. Its by-products like methyltriclosan and other chlorinated phenols pose a higher toxicity problem than the parent chemical itself (Dann and Hontela 2011). TCS is classified as not only a developmental toxicant but also as an endocrine disrupter. The effect of urinary TCS level and ovarian reserve was investigated, and it was found that urinary TCS concentration was inversely proportional to the antral follicle count (AFC). Urinary TCS level in women, which has shown a negative association with the AFC was found to be stronger in young and lean women (Mínguez-Alarcón et al. 2017). In a recent study, although similar negative association was reported with the AFC count, no significant alteration in the levels of FSH, AMH and estrogen was seen (Jurewicz et al. 2019). However, owing to the fact that AFC is also a potential factor contributing to ovarian reserve, TCS could act as a risk factor in diminishing the reserve.

Many EDCs act as ovarian steroidal agonists or antagonists and interfere in the steroidal metabolism. Therefore, they are considered inimical to the ovarian niche. BPA is an EDC and its interaction with ER is well established. Although it has weak affinity towards ER, its prolonged action has proved detrimental to the ovarian function (Vandenberg et al. 2009). BPA can reduce estradiol production which is one of the key features of POI (Mok-Lin et al. 2010). BPA is reported to increase the recruitment of follicles thereby causing a rapid decline in the follicular reserve. The exposure to BPA at any stage, be it prenatal, postnatal or adult stage, has shown to diminish the ovarian reserve of primordial follicles. A wealth of literature is available on the ovotoxic effects of BPA covering both preclinical and clinical studies. It was observed in a prospective cohort study that the urinary BPA concentrations of women with lower AFC undergoing treatment for infertility were high (Souter et al. 2013). Numerous preclinical studies have demonstrated the effect of phthalates in reducing the follicular reserve. Zhang et al*.*(2015) have shown that diethylhexyl phthalates (DEHP) has accelerated the follicular recruitment in the offspring mice at puberty when the maternal mice were exposed to DEHP during pregnancy. Interestingly, there was a rapid reduction in the primordial follicle pool even in the F2 generation of mice suggesting that the insufficiency due to DEHP can also be transgenerational. This can be a worrisome issue because, humans are exposed to phthalates on a daily basis, as they are very widely used in plastic and cosmetic industries. Another study reported an association between the urinary DEHP metabolite level with a decrease in AFC. The authors found that DEHP can adversely affect the pool of growing antral follicles and younger women appeared to be at a higher risk. Whether this diminishment of antral follicle pool was a result of decline in primordial follicles was not proven (Messerlian et al. 2016). Organophosphates, another class of EDCs, are also proven to have an adverse effect on ovarian reserve. Methoxychlor, an OP, has shown to diminish the follicular reserve, not only by inhibiting the ER expression, but also by epigenetic changes during the prenatal phase of follicular development in rats. Methoxychlor caused a significant reduction in the expression of ER beta which is predominantly present during all stages of follicle development on the granulosa cells. This study was allegedly the first to report that the compound promoted DNA hypermethylation by an increased expression of DNA methyl transferase in adult ovaries (Zama and Uzumcu 2009).

In a study by Park et al*.*(2019), the authors have reported the presence of volatile organic compounds such as toluene and xylene, and phthalates in synthetic plastic material used in the sanitary pads and diapers of certain brands. These materials are primarily used as liquid absorbents to improve the functionality of the products. It was reported that the quantity of these chemicals found in the products had a potential to cause abnormalities in the users. Among 11 brands tested, two of them contained significantly higher concentration of diethylhexyl phthalates (DEHP), than average of all the other sanitary pads. Also, every diaper brand tested contained di-n-butyl phthalate (DBP) and DEHP (Park et al. 2019). Considering the fact that sanitary pads and diapers are classified as hygiene products and their use is extremely common in women and children, more stringent regulations are essential. In fact, some studies have shown that DEHP can adversely affect the follicle pool. AFC was found to be reduced in women with detectable urinary DEHP metabolite and that young women were at a higher risk (Messerlian et al. 2016). Zhang et al*.*(2015) showed that the effects of DEHP in mice follicles were transgenerational.

Cigarette smoke, apart from posing a risk for PCOS, has also shown to significantly decline the ovarian reserve. Exposure to cigarette smoke in relation to diminishment of ovarian reserve, manifestation of POI and premature menopause was established very early in the literature (Harlow and Signorello 2000). PAHs are a group of compounds found in cigarette smoke. They are proven detrimental to the ovarian reserve in the offspring when exposed to the maternal mice (Jurisicova et al. 2007). Perhaps what makes PAHs more dangerous is that they exert their actions via AhR which plays a pivotal role right from the early stages of folliculogenesis. AhR has been widely studied for its role in reproduction, pregnancy and survival of fetus apart from its role in maintaining the dynamics of ovarian germ cells (Esteban et al. 2021; Abbott et al. 1999; Williams et al. 2014; Benedict et al. 2000). Apart from PAHs, persistent organic pollutants like 2,3,7,8-tetrachlorodibenzodioxin, dibenzofurans, dibenzo-p-dioxins and non-ortho substituted polychlorinated biphenyls can also act by AhRs (Vogel et al. 2020; Kawajiri and Fujii-Kuriyama^2017^; Khazaal et al. 2018). Possible mechanisms for the detrimental effects of cigarette smoke could be its propensity to raise the intracellular ROS and its action through AhR. Oxidative stress alters the intracellular calcium level in oocytes, and ROS can stimulate the OM causing untimely rupture of follicular cell (Kumar 2011). It is also evident that chronic stimulation of AhR by this group of compounds can cause premature senescence of ovaries (Shi et al. 2007; Hombach-Klonisch et al. 2012). PAHs are also present in particulate matter along with metals like copper, lead and zinc. Therefore, chronic exposure to air pollutants can also pose a risk of premature reproductive senescence. PM2.5 proved to be apoptotic in granulosa cells showing an increased oxidative stress (Agarwal et al. 2012; Tiwari et al. 2015; Liao et al. 2020).

Cadmium (Cd) is a heavy metal toxicant widely used in manufacturing batteries, metal smelting, municipal waste incineration etc. It is also found in cigarette smoke (Kumar and Sharma 2019)(ASTDR, 2012). In fact, rice, a staple food in most of the Asian countries is reported to be a major cause for Cd toxicity in non-smokers(Clemens et al. 2013; Song et al*.*^2017^). The levels of Cd in urine and blood are measurable, and this can be used to assess long-term or short-term exposures respectively, thereby making it possible to study the association between cadmium and various organ toxicities (Adams and Newcomb 2014). In granulosa cells, Cd is found to increase AMH, decrease KL (SCF) and induce apoptosis (Chen et al. 2020). Therefore, there is a possibility that Cd can affect the follicular development process by altering AMH levels. It was mentioned that since there is no value specified in guidelines for Cd levels pertaining to ovarian toxicity, the severity of effects needs to be established.

### Meiotic defects and aneuploidies

Meiosis, the basis for gametogenesis, is a remarkably conserved process in almost all eukaryotes. It is an extraordinary way of reducing the ploidy of the gametes in order to establish the ploidy of the species. Although meiosis happens in both the human gametes, spermatogenesis is simpler in terms of timeline without impeding the process intermittently. Conversely, oocyte meiosis is relatively perpetuated, more complex and occult. However, a significant progress is being made in unraveling the interposing processes. The process of meiosis, sometimes, can be erroneous and could result in aneuploidy. Aneuploidy is the presence of an abnormal number of chromosomes resulting from an erroneous meiosis. Aneuploidy can be of various types such as disomic, nullisomic, monosomic and trisomic resulting in preclinical abortions, spontaneous abortions, stillbirths or livebirths with defects. However, the most common aneuploidies involved in stillbirths and livebirths are either monosomic or trisomic (Hassold and Hunt 2001). Even if an aneuploidy perseveres in livebirths, they pose a high chance in manifesting some form of developmental disability as seen in trisomy 21 (Down’s syndrome). Perhaps, the complexity and impediment in oocytic meiosis might be the reason for the fact that most aneuploidies are a result of maternal meiotic errors, and it is not very intriguing that most of the trisomy 21 cases are of maternal origin. Furthermore, association was found between certain aneuploidies and some predisposing factors like parental origin (sex-specific), maternal age and environmental factors, and it appears to be hard-wired in the species (Hassold and Hunt 2001; Nagaoka et al. 2012). The mechanisms involved in different aneuploidies are explored in both meiosis I and II (MI and MII).

The effects of ETs on meiotic aneuploidies have long been studied. It is not till the twenty-first century that the researchers have found that ETs could potentially affect the gametes. It all started with an accidental discovery of chromosomal abnormalities induced by BPA in mice which was being used for meiotic studies inadvertently (Hunt et al. 2003). Since then, BPA has been extensively examined for its meiotic effects in vitro and in vivo in various stages of meiosis. BPA exposure was shown to increase the end-to-end associations and aberrations evident from recombination (Susiarjo et al. 2007). It affects meiosis in both early and late stages. In an in vitro study performed on human oocytes, it was observed that the degeneration of oocytes was higher in BPA-treated group, and an upregulation of certain genes resulted in double-stranded breaks (Brieno-Enriquez et al. 2012). This was supported by another study which showed that BPA could interfere with the repair mechanism involved in fixing the double-stranded breaks (Allard and Colaiácovo 2010). Another study by a group of scientists examined the acute, sub-chronic and chronic exposures of BPA on mice in which the chronic exposure group had premature separation of chromatids in MII (Pacchierotti et al. 2008). Many other studies proved the detrimental effects of BPA on chromosome. For instance, cytoskeleton aberrations, decreased bipolar spindle and altered synapses were seen with BPA exposure both in vitro and in vivo (Eichenlaub-Ritter et al. 2008; Machtinger et al. 2013). The effects of various ETs on meiosis are summarized in Table 1.

**Table 1:** Summary of the effect of various environmental toxicants on meiosis (MI and MII) at various stages

Agent	Effect	Source
BPA	Increased end-to-end associations and aberrations	Susiarjo et al. 2007
	Upregulation of genes involved in double-stranded breaks	Brieno-Enriquez et al. 2012, Allard et al. 2010
	Premature separation of chromatids in MII	Pacchierotti et al. 2008
	Cytoskeleton aberrations	Hunt et al. 2012
	Effect on oocyte maturation	Machtinger et al. 2013
	Decreased bipolar spindle	Machtinger et al. 2013
	Altered synapses	Susiarjo et al. 2007
	Disruption of genomic imprinting	Susiarjo et al. 2013
Increased maternal age	Decreased cohesion of sister chromatids	Nagaoka et al. 2012
	Missegregation of nonrecombinant bivalents	Perkins et al. 2016
TBO	Oocyte arrest at GV stage	Yang et al. 2019
	Misalignment of chromosomes	
Lead	Decreased rate of GVBD	Avazeri et al. 2006
Acrylamide	Abnormal spindle organization	Duan et al. 2015

It is well established that maternal age contributes immensely to meiotic aneuploidies, especially trisomies (Morton et al. 1988). The study was based on this hypothesis that the maternal age-dependent meiotic errors could be because of the accumulated ROS resulting from oxidative stress. As it was discussed earlier that the follicle pool established before the birth acts as a reserve throughout the reproductive span of a female, an increased oxidative stress can usher changes in the older oocytes paving way for premature cohesion loss and accumulating meiotic errors. Drosophila oocytes were used for the study, and conditional knock down of superoxide dismutase (SOD) in germline during midprophase I exhibited increased meiotic segregation errors. A decrease in the cohesion of sister chromatids and missegregation of nonrecombinant bivalents were reported (Perkins et al. 2016). These results also allow us to anticipate the role of ETs that promote long-term oxidative stress. Smoking has also been attributed as a cause of aneuploidies in female oocyte. Maternal smoking has been verily investigated as some aneuploidic causes. However, there have been contradictions regarding a direct association of maternal smoking especially because of confounding factors like maternal age (Chen et al. 1999). But it is not possible to completely rule out this risk factor since the role of oxidative stress has been well established in causing meiotic damages. In a population-based case-control study, mothers of livebirth Down’s syndrome were taken, and a questionnaire was used for analysis. Cases were split separately into cases having errors in MI and MII. When all the cases were analyzed for smoking as a risk factor, no evidence of association was found. But when MI and MII were assessed separately, a decrease in the risk in MI and increase in risk in MII were observed specifically with younger mothers (Yang 1999). In another study, nicotine could affect the meiotic spindle in MI interfering with the OM in cultured mouse oocytes. Abnormal metaphase II with aberrant spindles and unaligned chromosomes were reported, although GV to MI transition was normal (Zenzes and Bielecki 2004). Another chemical, tributylene oxide (TBO), which is extensively used in plastic industries as a stabilizer apart from other uses, was also studied on cultured mouse oocytes, where it was seen to arrest the oocytes at GV stage and misalign the chromosomes. A buildup of oxidative stress with elevated ROS, higher rate of aneuploidies in treated group and even apoptosis of oocytes were the other features that were observed (Yang et al. 2019).

In a first-time report of lead toxicity on oocyte meiosis, it was revealed that lead can affect meiosis in mouse oocyte in vitro at a concentration as low as femtomolar (fM). The rate of germinal vesicle breakdown (GVBD) was examined both in cumulus-enclosed and cumulus-free oocytes and the rate of GVBD of cumulus-enclosed oocytes was found to be reduced significantly with 10fM Pb(NO_3_)_2_ but unaltered in cumulus-free oocytes (Avazeri et al. 2006). Acrylamide (ACD) is a highly toxic organic agent used as a thickening agent in polymer manufacturing industries and as a flocculant agent in water treatment. When studied in mice oocytes in vivo, abnormal spindle organization was reported with disrupted oocytes thereby affecting the oocyte development competence. In the ACD-treated group, a large proportion of oocytes remained in the GV stage compared to the cells of control group. ACD could also alter the transcriptional activity of the genome which was observed by the significant reduction in H3K27me3 expression (Duan et al. 2015). In spite of abundance of data, there is still no direct risk that is established for aneuploidy with ETs, except in case of BPA. Effects of ETs on different stages of MI and MII are tabulated in Table 1. However, it is clearly understood that ETs’ association with meiotic errors is not something that can be ignored given the fact that chronic exposure can have deleterious effects and could go unnoticed under the heels of other quintessential factors.

## Conclusion

We have made a considerable progress in drawing substantial experimental evidence with respect to the detrimental effects of ETs on female fertility. Nevertheless, there is still an ocean of things to explore so as to understand the molecular mechanisms involved. Although the effects of toxicants like BPA and phthalates have been extensively studied, there may be many other toxicants that persist in the environment, the upshots of which remain unprecedented and unexplored till date. For instance, determining the effect of ETs on meiosis remains obscure owing to the fact that either the aftermath is hard to detect or their influence is often neglected. Similarly, it is difficult to establish the role of a single ET as a risk factor in conditions like PCOS and POI where multiple causative factors are involved. Understanding the implications of ETs on other facets like increase-in-time-to-pregnancy and idiopathic infertility is crucial. Therefore, reliable models that eliminate confounding factors and create well-founded evidence are crucial. Many more epidemiological studies in addition to experimental research are pivotal, and such studies must be encouraged. Projects like ‘European Human Biomonitoring Initiative’ (Hbm4eu.eu 2021) that work to generate knowledge from the data based on scientific studies in order to manage the use of chemicals for protection of human health has been releasing the list of high priority substances in the 5-year project (2016–2021). Such initiaives are essential not only in one part but throughout the world. High priority substance lists released by the project include substances like bisphenol, PAHs, per/poly-fluorinated compounds, heavy metals like arsenic, cadmium, chromium, lead and mercury, pesticides, acrylamide (EEA 2018; Hmb4eu 2021). Another important aspect which needs immediate attention is that the consequences of ETs on reproductive physiology are not only evident in humans, but also in wildlife. For example, the occurrence of MOF is well established in alligators, lambs, monkeys, etc. Most of these ETs are anthropogenic and can be controlled. Therefore, the repercussions pertaining to ETs on fertility are not to be treated as isolated cases but should be considered as a matter of public and wildlife health. The evidence being generated from research must be used to reform the existing environment policies.

## Data Availability

Not applicable.

## References

[CR1] Abbott BD, Schmid JE, Pitt JA (1999). Adverse reproductive outcomes in the transgenic Ah receptor-deficient mouse. Toxicol Appl Pharmacol.

[CR2] Abbott DH, Barnett DK, Bruns CM, Dumesic DA (2005). Androgen excess fetal programming of female reproduction: a developmental aetiology for polycystic ovary syndrome?. Hum Reprod Update.

[CR3] Adams SV, Newcomb PA (2014). Cadmium blood and urine concentrations as measures of exposure: NHANES 1999-2010. J Expo Sci Environ Epidemiol.

[CR4] Agarwal A, Aponte-Mellado A, Premkumar BJ (2012). The effects of oxidative stress on female reproduction: a review. Reprod Biol Endocrinol.

[CR5] Allard P, Colaiácovo MP (2010). Bisphenol A impairs the double-strand break repair machinery in the germline and causes chromosome abnormalities. Proc Natl Acad Sci U S A.

[CR6] Armenti AME, Zama AM, Passantino L, Uzumcu M (2008). Developmental methoxychlor exposure affects multiple reproductive parameters and ovarian folliculogenesis and gene expression in adult rats. Toxicol Appl Pharmacol.

[CR7] Ashley DJB (1973) Origin of teratomas. Cancer 32:390–394. 10.1002/1097-0142(197308)32:2<390::AID-CNCR2820320216>3.0.CO;2-W 10.1002/1097-0142(197308)32:2<390::aid-cncr2820320216>3.0.co;2-w 4722920

[CR8] ASTDR, Agency for toxic substances and disease agency (2012) Toxicological profiles. https://www.atsdr.cdc.gov/toxprofiles/tp5.pdf. Accessed on 25 May 2021.

[CR9] Avazeri N, Denys A, Lefèvre B (2006). Lead cations affect the control of both meiosis arrest and meiosis resumption of the mouse oocyte in vitro at least via the PKC pathway. Biochimie.

[CR10] Azziz R, Carmina E, Dewailly D (2009). The Androgen Excess and PCOS Society criteria for the polycystic ovary syndrome: the complete task force report. Fertil Steril.

[CR11] Azziz R, Sanchez LA, Knochenhauer ES (2004). Androgen excess in women: experience with over 1000 consecutive patients. J Clin Endocrinol Metab.

[CR12] Barker DJP (2003). The developmental origins of adult disease. Eur J Epidemiol.

[CR13] Beischlag TV, Morales JL, Hollingshead BD, Perdew GH (2008). The aryl hydrocarbon receptor complex and the control of gene expression. Crit Rev Eukaryot Gene Expr.

[CR14] Benedict JC, Lin T-M, Loeffler IK, et al. (2000) Physiological role of the aryl hydrocarbon receptor in mouse ovary development. Toxicol Sci 6(2):382-8. doi: 10.1093/toxsci/56.2.382. 10.1093/toxsci/56.2.382 10910997

[CR15] Brieno-Enriquez MA, Reig-Viader R, Cabero L (2012). Gene expression is altered after bisphenol A exposure in human fetal oocytes in vitro. Mol Hum Reprod.

[CR16] Bristol-Gould SK, Kreeger PK, Selkirk CG (2006). Postnatal regulation of germ cells by activin: the establishment of the initial follicle pool. Dev Biol.

[CR17] Broekmans FJ, Knauff EAH, Valkenburg O (2006). PCOS according to the Rotterdam consensus criteria: change in prevalence among WHO-II anovulation and association with metabolic factors. BJOG An Int J Obstet Gynaecol.

[CR18] Chen C-L, Gilbert TJ, Daling JR (1999). Maternal smoking and Down syndrome: the confounding effect of maternal age. Am J Epidemiol.

[CR19] Chen N, Luo L, Zhang C (2020). Anti-Müllerian hormone participates in ovarian granulosa cell damage due to cadmium exposure by negatively regulating stem cell factor. Reprod Toxicol.

[CR20] Chen Y, Jefferson WN, Newbold RR, Pepling MK (2007). Estradiol, progesterone, and genistein inhibit oocyte nest breakdown and primordial follicle assembly in the neonatal mouse ovary in vitro and in vivo. Endocrinology.

[CR21] Clemens S, Aarts MGM, Thomine S, Verbruggen N (2013). Plant science: the key to preventing slow cadmium poisoning. Trends Plant Sci.

[CR22] Cohn BA, Cirillo PM, Wolff MS (2003). DDT and DDE exposure in mothers and time to pregnancy in daughters. Lancet.

[CR23] Couse JF, Yates MM, Deroo BJ, Korach KS (2005). Estrogen receptor-β is critical to granulosa cell differentiation and the ovulatory response to gonadotropins. Endocrinology.

[CR24] Craig ZR, Wang W, Flaws JA (2011). Endocrine-disrupting chemicals in ovarian function: effects on steroidogenesis, metabolism and nuclear receptor signaling. Reproduction.

[CR25] Dann AB, Hontela A (2011). Triclosan: environmental exposure, toxicity and mechanisms of action. J Appl Toxicol.

[CR26] De Felip E, Di Domenico A, Miniero R, Silvestroni L (2004). Polychlorobiphenyls and other organochlorine compounds in human follicular fluid. Chemosphere.

[CR27] De Vos M, Devroey P, Fauser BC (2010). Primary ovarian insufficiency. Lancet.

[CR28] Diamanti-Kandarakis E, Bourguignon JP, Giudice LC (2009). Endocrine-disrupting chemicals: an Endocrine Society scientific statement. Endocr Rev.

[CR29] Ding N, Harlow SD, Randolph JF (2020). Perfluoroalkyl and polyfluoroalkyl substances (PFAS) and their effects on the ovary. Hum Reprod Update.

[CR30] Dominguez MA, Sadeu JC, Guerra MT et al (2016) Ovarian toxicity of environmental contaminants: 50 shades of grey. Mol Integr Toxicol:215–244. 10.1007/978-3-319-27449-2_7

[CR31] Duan X, Wang Q-C, Chen K-L (2015). Acrylamide toxic effects on mouse oocyte quality and fertility in vivo. Sci Rep.

[CR32] Edwards DA (1971). Neonatal administration of androstenedione, testosterone or testosterone propionate: effects on ovulation, sexual receptivity and aggressive behavior in female mice. Physiol Behav.

[CR33] Eichenlaub-Ritter U, Vogt E, Cukurcam S (2008). Exposure of mouse oocytes to bisphenol A causes meiotic arrest but not aneuploidy. Mutat Res Genet Toxicol Environ Mutagen.

[CR34] Esteban J, Sánchez-Pérez I, Hamscher G (2021). Role of aryl hydrocarbon receptor (AHR) in overall retinoid metabolism: response comparisons to 2,3,7,8-tetrachlorodibenzo-p-dioxin (TCDD) exposure between wild-type and AHR knockout mice. Reprod Toxicol.

[CR35] Forabosco A, Sforza C (2007). Establishment of ovarian reserve: a quantitative morphometric study of the developing human ovary. Fertil Steril.

[CR36] Fowler PA, Anderson RA, Saunders PT (2011). Development of steroid signaling pathways during primordial follicle formation in the human fetal ovary. J Clin Endocrinol Metab.

[CR37] Gallo MV, Ravenscroft J, Carpenter DO (2016). Endocrine disrupting chemicals and ovulation: is there a relationship?. Environ Res.

[CR38] Gonsioroski A, Mourikes VE, Flaws JA (2020) Endocrine disruptors in water and their effects on the reproductive system. Int J Mol Sci 2020, Vol 21, Page 1929 21:1929. 10.3390/IJMS21061929 10.3390/ijms21061929 PMC7139484 32178293

[CR39] Gunnarsson D, Leffler P, Ekwurtzel E (2008). Mono-(2-ethylhexyl) phthalate stimulates basal steroidogenesis by a cAMP-independent mechanism in mouse gonadal cells of both sexes. Reproduction.

[CR40] Harlow BL, Signorello LB (2000). Factors associated with early menopause. Maturitas.

[CR41] Hassold T, Hunt P (2001). To err (meiotically) is human: the genesis of human aneuploidy. Nat Rev Genet.

[CR42] HBM4EU priority substances and the prioritisation strategy—European Environment Agency. https://www.eea.europa.eu/themes/human/human-biomonitoring/prioritisation-and-substances. Accessed 2 Sep 2021

[CR43] Hmb4u.eu About HBM4EU | HBM4EU—science and policy for a healthy future. https://www.hbm4eu.eu/about-hbm4eu/. Accessed 2 Sep 2021

[CR44] Hombach-Klonisch S, Pocar P, Kietz S, Klonisch T (2012). Molecular actions of polyhalogenated arylhydrocarbons (PAHs) in female reproduction. Curr Med Chem.

[CR45] Huang A, Brennan K, Azziz R (2010). Prevalence of hyperandrogenemia in the polycystic ovary syndrome diagnosed by the National Institutes of Health 1990 criteria. Fertil Steril.

[CR46] Hughesdon PE (1982) Morphology and morphogenesis of the stein-leventhal ovary and of so-called “Hyperthecosis.” Obstet Gynecol Surv 37:59–77. 10.1097/00006254-198202000-00001 10.1097/00006254-198202000-00001 7033852

[CR47] Hunt PA, Koehler KE, Susiarjo M (2003). Bisphenol a exposure causes meiotic aneuploidy in the female mouse. Curr Biol.

[CR48] Hunt PA, Lawson C, Gieske M (2012). Bisphenol A alters early oogenesis and follicle formation in the fetal ovary of the rhesus monkey. Proc Natl Acad Sci.

[CR49] Johansson HKL, Svingen T, Fowler PA (2017). Environmental influences on ovarian dysgenesis—developmental windows sensitive to chemical exposures. Nat Rev Endocrinol.

[CR50] Jurewicz J, Wielgomas B, Radwan M (2019). Triclosan exposure and ovarian reserve. Reprod Toxicol.

[CR51] Jurisicova A, Taniuchi A, Li H (2007). Maternal exposure to polycyclic aromatic hydrocarbons diminishes murine ovarian reserve via induction of Harakiri. J Clin Invest.

[CR52] Kandaraki E, Chatzigeorgiou A, Livadas S (2011). Endocrine disruptors and Polycystic Ovary Syndrome (PCOS): elevated serum levels of bisphenol A in women with PCOS. J Clin Endocrinol Metab.

[CR53] Kawajiri K, Fujii-Kuriyama Y (2017). The aryl hydrocarbon receptor: a multifunctional chemical sensor for hostdefense and homeostatic maintenance. Exp Anim.

[CR54] Khazaal AQ, Jaeger CD, Bottum KM, Tischkau SA (2018). Environmental factors act through aryl hydrocarbon receptor activation and circadian rhythm disruption to regulate energy metabolism. J Receptor Ligand Channel Res.

[CR55] Kimura F, Bonomi LM, Schneyer AL (2011). Follistatin regulates germ cell nest breakdown and primordial follicle formation. Endocrinology.

[CR56] Kipp JL, Kilen SM, Bristol-Gould S (2007). Neonatal exposure to estrogens suppresses activin expression and signaling in the mouse ovary. Endocrinology.

[CR57] Knauff EAH, Eijkemans MJC, Lambalk CB (2009). Anti-Müllerian hormone, inhibin b, and antral follicle count in young women with ovarian failure. J Clin Endocrinol Metab.

[CR58] Kumar S (2018). Occupational and environmental exposure to lead and reproductive health impairment: an overview. Indian J Occup Environ Med.

[CR59] Kumar S (2011). Occupational, environmental and lifestyle factors associated with spontaneous abortion. Reprod Sci.

[CR60] Kumar S, Sharma A (2019) Cadmium toxicity: effects on human reproduction and fertility. Rev Environ Health 34. 10.1515/REVEH-2019-0016 10.1515/reveh-2019-0016 31129655

[CR61] La Merrill MA, Vandenberg LN, Smith MT (2020). Consensus on the key characteristics of endocrine-disrupting chemicals as a basis for hazard identification. Nat Rev Endocrinol.

[CR62] Lee S, Min J, Min K (2020) Female infertility associated with blood lead and cadmium levels. Int J Environ Res Public Health:17. 10.3390/IJERPH17051794 10.3390/ijerph17051794 PMC7084729 32164251

[CR63] Liao B-Q, Liu C-B, Xie S-J (2020). Effects of fine particulate matter (PM2.5) on ovarian function and embryo quality in mice. Environ Int.

[CR64] Lin S-Y, Yang Y-C, Chang CY-Y (2019). Risk of polycystic ovary syndrome in women exposed to fine air pollutants and acidic gases: a nationwide cohort analysis. Int J Environ Res Public Health.

[CR65] Lookingland KJ, Wise PM, Barraclough CA (1982). Failure of the hypothalamic noradrenergic system to function in adult androgen-sterilized rats. Biol Reprod.

[CR66] Luderer U (2014). Ovarian toxicity from reactive oxygen species.

[CR67] Machtinger R, Combelles CMH, Missmer SA (2013). Bisphenol-A and human oocyte maturation in vitro. Hum Reprod.

[CR68] McDonald PG, Doughty C (1972). Comparison of the effect of neonatal administration of testosterone and dihydrotestosterone in the female rat. J Reprod Fertil.

[CR69] Meeker JD, Missmer SA, Altshul L (2009). Serum and follicular fluid organochlorine concentrations among women undergoing assisted reproduction technologies. Environ Health.

[CR70] Messerlian C, Souter I, Gaskins AJ (2016). Urinary phthalate metabolites and ovarian reserve among women seeking infertility care. Hum Reprod.

[CR71] Mínguez-Alarcón L, Christou G, Messerlian C (2017). Urinary triclosan concentrations and diminished ovarian reserve among women undergoing treatment in a fertility clinic. Fertil Steril.

[CR72] Mok-Lin E, Ehrlich S, Williams PL (2010). Urinary bisphenol A concentrations and ovarian response among women undergoing IVF. Int J Androl.

[CR73] MORTON NE, JACOBS PA, HASSOLD T, WU D (1988) Maternal age in trisomy. Ann Hum Genet 52:227–235. 10.1111/j.1469-1809.1988.tb01100.x 10.1111/j.1469-1809.1988.tb01100.x 2977936

[CR74] Motta PM, Makabe S, Nottola SA (1997). The ultrastructure of human reproduction. 1. The natural history of the female germ cell: origin, migration and differentiation inside the developing ovary. Hum Reprod Update.

[CR75] Muretto P, Chilosi M, Rabitti C (2001). Biovularity and “coalescence of primary follicles” in ovaries with mature teratomas. Int J Surg Pathol.

[CR76] Nagaoka SI, Hassold TJ, Hunt PA (2012). Human aneuploidy: mechanisms and new insights into an age-old problem. Nat Rev Genet.

[CR77] Nelson VL, Qin KN, Rosenfield RL (2001). The biochemical basis for increased testosterone production in theca cells propagated from patients with polycystic ovary syndrome. J Clin Endocrinol Metab.

[CR78] Massányi P, Massányi M, Madeddu R, Stawarz R, Lukáč N (2020) Effects of Cadmium, Lead, and Mercury on the Structure and Function of Reproductive Organs. Toxics 8(4):94; 1-31. 10.3390/toxics8040094 10.3390/toxics8040094 PMC7711607 33137881

[CR79] Pacchierotti F, Ranaldi R, Eichenlaub-Ritter U (2008). Evaluation of aneugenic effects of bisphenol A in somatic and germ cells of the mouse. Mutat Res Genet Toxicol Environ Mutagen.

[CR80] Palacio OD, Triana O, Gaviria A (2006). Autosomal microsatellite data from Northwestern Colombia. Forensic Sci Int.

[CR81] Park CJ, Barakat R, Ulanov A (2019). Sanitary pads and diapers contain higher phthalate contents than those in common commercial plastic products. Reprod Toxicol.

[CR82] Pepling ME, Spradling AC (1998). Female mouse germ cells form synchronously dividing cysts. Development.

[CR83] Pepling ME, Spradling AC (2001). Mouse ovarian germ cell cysts undergo programmed breakdown to form primordial follicles. Dev Biol.

[CR84] Perkins AT, Das TM, Panzera LC, Bickel SE (2016). Oxidative stress in oocytes during midprophase induces premature loss of cohesion and chromosome segregation errors. Proc Natl Acad Sci U S A.

[CR85] Pru JK, Kaneko-Tarui T, Jurisicova A (2009). Induction of proapoptotic gene expression and recruitment of p53 herald ovarian follicle loss caused by polycyclic aromatic hydrocarbons. Reprod Sci.

[CR86] Puurunen J, Piltonen T, Jaakkola P (2009). Adrenal androgen production capacity remains high up to menopause in women with polycystic ovary syndrome. J Clin Endocrinol Metab.

[CR87] Ragusa A, Svelato A, Santacroce C (2021). Plasticenta: first evidence of microplastics in human placenta. Environ Int.

[CR88] Rivera OE, Varayoud J, Rodríguez HA (2011). Neonatal exposure to bisphenol A or diethylstilbestrol alters the ovarian follicular dynamics in the lamb. Reprod Toxicol.

[CR89] Robles R, Morita Y, Mann KK (2000). The aryl hydrocarbon receptor, a basic helix-loop-helix transcription factor of the PAS gene family, is required for normal ovarian germ cell dynamics in the mouse. Endocrinology.

[CR90] Rosenfield RL, Ehrmann DA (2016). The pathogenesis of polycystic ovary syndrome (PCOS): the hypothesis of PCOS as functional ovarian hyperandrogenism revisited. Endocr Rev.

[CR91] Rutkowska AZ, Diamanti-Kandarakis E (2016). Polycystic ovary syndrome and environmental toxins. Fertil Steril.

[CR92] Shanle EK, Xu W (2011). Endocrine disrupting chemicals targeting estrogen receptor signaling: Identification and mechanisms of action. Chem Res Toxicol.

[CR93] Shi Z, Valdez KE, Ting AY (2007). Ovarian endocrine disruption underlies premature reproductive senescence following environmentally relevant chronic exposure to the aryl hydrocarbon receptor agonist 2,3,7,8-tetrachlorodibenzo-p-dioxin. Biol Reprod.

[CR94] Silva-Santos KCMMS, Seneda MM, Silva-Santos KC, Seneda MM (2018). Multioocyte follicles in adult mammalian ovaries. Anim Reprod.

[CR95] Song Y, Wang Y, Mao W, Sui H, Yong L, Yang D, Jiang D, Zhang L, Gong Y (2017) Dietary cadmium exposure assessment among the Chinese population. Plos One. 10.1371/journal.pone.0177978 10.1371/journal.pone.0177978 PMC5436861 28542445

[CR96] Souter I, Smith KW, Dimitriadis I (2013). The association of bisphenol-A urinary concentrations with antral follicle counts and other measures of ovarian reserve in women undergoing infertility treatments. Reprod Toxicol.

[CR97] Stankiewicz T, Błaszczyk B, Udała J (2009). A study on the occurrence of polyovular follicles in porcine ovaries with particular reference to intrafollicular hormone concentrations, quality of oocytes and their in vitro fertilization. J Vet Med Ser C Anat Histol Embryol.

[CR98] Susiarjo M, Hassold TJ, Freeman E, Hunt PA (2007). Bisphenol A exposure in utero disrupts early oogenesis in the mouse. PLoS Genet.

[CR99] Suzuki A, Sugihara A, Uchida K (2002). Developmental effects of perinatal exposure to bisphenol-A and diethylstilbestrol on reproductive organs in female mice. Reprod Toxicol.

[CR100] Takeuchi T, Tsutsumi O, Ikezuki Y (2004). Positive relationship between androgen and the endocrine disruptor, bisphenol A, in normal women and women with ovarian dysfunction. Endocr J.

[CR101] Telfer E, Gosden RG (1987). A quantitative cytological study of polyovular follicles in mammalian ovaries with particular reference to the domestic bitch (Canis familiaris). J Reprod Fertil.

[CR102] Thau R, Lanman JT, Brinson A (1976). Declining plasma progesterone concentration with advancing gestation in blood from umbilical uterine veins and fetal heart in monkeys. Biol Reprod.

[CR103] Tiwari M, Prasad S, Tripathi A (2015). Apoptosis in mammalian oocytes: a review. Apoptosis.

[CR104] Tokmak A, Yıldırım G, Sarıkaya E (2015). Increased oxidative stress markers may be a promising indicator of risk for primary ovarian insufficiency: a cross-sectional case control study. Rev Bras Ginecol e Obs.

[CR105] Toxicological profiles. Agency for toxic substances and disease agency. https://www.atsdr.cdc.gov/toxprofiles/tp5.pdf. Accessed on 25 May 2021.

[CR106] Vabre P, Gatimel N, Moreau J (2017). Environmental pollutants, a possible etiology for premature ovarian insufficiency: a narrative review of animal and human data. Environ Heal A Glob Access Sci Source.

[CR107] Vagi SJ, Azziz-Baumgartner E, Sjödin A (2014). Exploring the potential association between brominated diphenyl ethers, polychlorinated biphenyls, organochlorine pesticides, perfluorinated compounds, phthalates, and bisphenol a in polycystic ovary syndrome: a case-control study. BMC Endocr Disord.

[CR108] Valgeirsdottir H, Vanky E, Sundström-Poromaa I (2019). Prenatal exposures and birth indices, and subsequent risk of polycystic ovary syndrome: a national registry-based cohort study. BJOG An Int J Obstet Gynaecol.

[CR109] Vandenberg LN, Maffini MV, Sonnenschein C (2009). Bisphenol-a and the great divide: a review of controversies in the field of endocrine disruption. Endocr Rev.

[CR110] Vélez MP, Arbuckle TE, Fraser WD (2015). Maternal exposure to perfluorinated chemicals and reduced fecundity: the MIREC study. Hum Reprod.

[CR111] Victor VM, Rocha M, Bañuls C (2011). Induction of oxidative stress and human leukocyte/endothelial cell interactions in polycystic ovary syndrome patients with insulin resistance. J Clin Endocrinol Metab.

[CR112] Vogel CFA, Van Winkle LS, Esser C, Haarmann-Stemmann T (2020) The aryl hydrocarbon receptor as a target of environmental stressors—implications for pollution mediated stress and inflammatory responses. Redox Biol 34:101530. 10.1016/J.REDOX.2020.101530 10.1016/j.redox.2020.101530 PMC7327980 32354640

[CR113] Wear HM, McPike MJ, Watanabe KH (2016). From primordial germ cells to primordial follicles: a review and visual representation of early ovarian development in mice. J Ovarian Res.

[CR114] Wee SY, Aris AZ (2019) Occurrence and public-perceived risk of endocrine disrupting compounds in drinking water. npj Clean Water 2:4. 10.1038/s41545-018-0029-3

[CR115] Weil S, Vendola K, Zhou J, Bondy CA (1999). Androgen and follicle-stimulating hormone interactions in primate ovarian follicle development. J Clin Endocrinol Metab.

[CR116] Welt CK (2008). Primary ovarian insufficiency: a more accurate term for premature ovarian failure. Clin Endocrinol.

[CR117] Williams EG, Mouchiroud L, Frochaux M (2014). An evolutionarily conserved role for the aryl hydrocarbon receptor in the regulation of movement. PLoS Genet.

[CR118] Willis DS, Watson H, Mason HD (1998). Premature response to luteinizing hormone of granulosa cells from anovulatory women with polycystic ovary syndrome: relevance to mechanism of anovulation 1. J Clin Endocrinol Metab.

[CR119] Wójtowicz AK, M K, EŁ G (2007). DDT- and DDE-induced disruption of ovarian steroidogenesis in prepubertal porcine ovarian follicles: a possible interaction with the main steroidogenic enzymes and estrogen receptor beta. J Physiol Pharmacol.

[CR120] Woodruff TK, Mayo KE (2005). To β or not to β: estrogen receptors and ovarian function. Endocrinology.

[CR121] Yang LL, Cui YX, Ma JY (2019). Tributyltin oxide exposure impairs mouse oocyte maturation and its possible mechanisms. J Cell Biochem.

[CR122] Yang Q (1999). Risk factors for trisomy 21: maternal cigarette smoking and oral contraceptive use in a population-based case-control study. Genet Med.

[CR123] Yang Q, Zhao Y, Qiu X (2015). Association of serum levels of typical organic pollutants with polycystic ovary syndrome (PCOS): a case-control study. Hum Reprod.

[CR124] Yatsenko SA, Rajkovic A (2019). Genetics of human female infertility. Biol Reprod.

[CR125] Zachos NC, Billiar RB, Albrecht ED, Pepe GJ (2004). Regulation of oocyte microvilli development in the baboon fetal ovary by estrogen. Endocrinology.

[CR126] Zama AM, Uzumcu M (2009). Fetal and neonatal exposure to the endocrine disruptor methoxychlor causes epigenetic alterations in adult ovarian genes. Endocrinology.

[CR127] Zenzes M, Bielecki R (2004). Nicotine-induced disturbances of meiotic maturation in cultured mouse oocytes: alterations of spindle integrity and chromosome alignment. Tob Induc Dis.

[CR128] Zhang B, Zhang B, Zhang B (2020). Lifestyle and environmental contributions to ovulatory dysfunction in women of polycystic ovary syndrome. BMC Endocr Disord.

[CR129] Zhou W, Liu J, Liao L, Han S, Liu J (2008) Effect of bisphenol A on steroid hormone production in rat ovarian theca-interstitial and granulosa cells. Mol Cell Endocrinol. 283(1-2):12–8. 10.1016/j.mce.2007.10.010 10.1016/j.mce.2007.10.010 18191889

